# A social work contribution in end-of-life care: incorporating critical spirituality

**DOI:** 10.1177/26323524241282683

**Published:** 2024-09-30

**Authors:** Fiona Gardner

**Affiliations:** Social Work, La Trobe Rural Health School, La Trobe University, Edwards Road, Bendigo, VIC 3552, Australia

**Keywords:** critical reflection, critical spirituality, palliative care

## Abstract

This article outlines how a framework for critical spirituality incorporated into a social work perspective, can contribute to work in end-of-life care. This is based on my experience of working in interdisciplinary teams, including pastoral care workers or chaplains, nurses, doctors, a range of carers and other allied health professionals. Traditionally, social workers have focused on the holistic well-being of the dying person and their families but tended not to actively include the spiritual. However, there is increased recognition in social work of the value of integrating critical spirituality: understanding the person’s own sense of meaning and the social assumptions that might influence how this can be expressed. This might mean working with individuals and families on understanding how societal expectations of the dying process are undermining the person’s desire to die in a particular way. To do this, workers must themselves recognise their own internalised societal assumptions and be willing to challenge these. The article explores the potential value of how such a social work approach can integrate critical, postmodern, green and relational theories as well as indigenous worldviews with key qualities of practice such as humility, deep listening and waiting, and the ability to be critically reflective. Key ideas are used to help focus practice and the inclusion of critical spirituality. These include (1) exploring the influence of the person’s history and social context; (2) the value of particular relationships and networks, including community and environmental connections and activities and processes that are an intrinsic aspect of the person’s well-being; (3) challenging workers to be critically and reflectively aware of their own assumptions and values to ensure the dying person can truly express their preferences related to death and dying. Examples from my experience are used to illuminate how such perspectives can be actively included in practice across professional boundaries to shift perceptions of ‘how things are done here’ to what can be more life enhancing for those in palliative care.

I had a friend I will call Cathy who died recently in a palliative care wing in a hospital. When I was writing this article, I became conscious that her story could be adapted (and anonymised) to illustrate how social workers in palliative care could use critical spirituality to benefit those they worked with and their colleagues. Once Cathy was confident that she was not likely to die in pain, what she really wanted, when she had the energy, was to talk about what had made her life meaningful. From one perspective, her life could seem like a series of undermining tragedies, but for her what stood out was a life of connections. She still struggled to make sense of some experiences and she often returned to these wanting to see them in a more life and death-enhancing way. This particularly related to an experience of being made redundant from an organisation auspiced by the religious tradition she shared. Cathy would have named this exploration as a spiritual process because the spiritual (and religious) was explicitly important to her. Others might see this as coming to terms with their life’s journey or becoming reconciled to the key concerns of existence ‘death, isolation, meaning in life and freedom’.^1^ In this particular palliative care team in Victoria, Australia, Cathy had an interdisciplinary palliative care team, including nurses, attendant carers, a physiotherapist, a music therapist and a pastoral care worker. No social worker (apart from my visits). In another palliative care organisation, Cathy might have had a social worker. This article explores what might have been possible if a social worker had been trained in a critical spirituality perspective and had been involved with Cathy and her team.

Exactly what we mean by the spiritual can be hard to define^2^). In seeking a shared understanding, an International Conference on Palliative Care defined spirituality as

the aspect of humanity that refers to the way individuals seek and express meaning and purpose, and the way they experience their connectedness to the moment, to self, to others, to nature and to the significant or sacred.^3^

It is important to affirm that for some of those involved in palliative care spirituality includes a religious tradition with a fundamental influence on how they have lived and implications for dying and death.^4^ People may not seek services because they fear their religious beliefs will not be understood or respected.^5^ Workers may be reluctant to explore religious beliefs feeling they don’t ‘know enough’ or do not understand that each individual or family will experience their religious beliefs from their own perspective.^6^ It helps to move beyond the apparent duality of spirituality and religion to see how both can be about the experience of transcendental and fundamental interconnectedness.^7^

Social work’s relationship to spirituality and religion has shifted significantly in the last 20 years. Initially, the social work profession was underpinned by religious traditions, but shifted in the 1950s to being essentially secular, at least in the West, because of concerns about religious proselytising.^8^ The latest International Federation of Social Work code of ethics reflects current thinking and includes challenging discrimination related to religion or spiritual beliefs.^9^ There are also rapidly increasing numbers of articles and books related to including spirituality and religion in practice such as Crisp’s *Handbook of Religion, Spirituality and Social Work*.^2^ Similarly in palliative care, there has been a shift from a fairly minimal mention of spiritual care in social work^10^ perhaps because spiritual care was seen as a separate discipline^11^ or that palliative care social workers saw their role as emphasising the socio-cultural^12^ and developmental^13^ or practical^14^ with the spiritual linked to culture rather than to having significance in itself and potential for community support.^15^

Social work research in palliative care has addressed the qualities seen as desirable much of which implicitly suggests aspects of spiritual care. Beresford et al.^10^ found that service users in palliative care wanted genuine friendship, meaning some reciprocity in relationships and willingness to share feelings, both sadness and laughter, caring and nuanced qualities, including the capacity to listen with an accepting, non-judgemental attitude. Service users also valued ‘having a sense of control. . . a real say in the process’ which is less contentious in palliative care where ‘there is no statutory requirement to make use of palliative care services, so there is a sense in which the service user remains in control of the relationship and can opt out if they choose’.^16^

However, there is growing recognising that social workers also need to more actively recognise the importance of faith as well as spirituality.^17^ This is particularly for those who are dying, seeing ‘clients’ religious and spiritual commitments as an ally in the process of strengthening social connections’^18^ particularly in times of death anxiety. Social workers traditionally have had little training in spirituality, but Rezenbrink^19^ points out that social workers in end-of-life care are asked ‘to enter into places of pain and to respond with compassion and wisdom based on their professional and person life experiences’. She adds that this is challenging work; support and skilled supervision are needed as well as something for the soul of the worker. More recent Canadian research suggests social worker teams want to work on ‘nonphysical suffering’ including wrestling with the issues of meaning and faith, but lack time: practical needs take over combined with a struggle to manage their own and others’ expectations they can relieve spiritual suffering^20^ rather than developing the capacity to sit with helplessness as connectedness.^21^ Social workers’ may also experience tensions between their preferred nuanced and critical professional responses and the more measurable outcomes expected by their employing organisation.^7^

Typically, palliative care services in Australia include spirituality as part of holistic care as well as psychological and social support.^22^ This is usually provided through an interdisciplinary team that *may* include, as well as nursing and medical support, social workers in such roles as family support and loss and grief, other allied health staff and volunteers. However, teams do not necessarily have a social worker. The makeup of the particular team will depend on the particular organisation and its preferences and the availability of staff. Many palliative care services, though not all, would provide a spiritual support worker, who might be called a chaplain, pastoral care worker or well-being worker as well as volunteers. When members of palliative care teams in several locations in Victoria, Australia were asked who should be responsible for including the spiritual or religious in palliative care, they almost always said everyone in the team.^23^ They articulated that as they are dying, people still want to engage with what is meaningful to them and for at least some, to explore the fundamental questions of life: who am I now?; what is and has my life been about? However, for many of those who are seriously ill, access to those from a religious tradition is also important.^24^

The question then becomes who is it that people want to talk with? Is it a specialist spiritual care worker, a social worker or might it be anyone on the team? Clearly, there are times when individuals want a specialist, including someone from a religious tradition and the team needs to know when to refer.^7^ However, it isn’t always possible to predict when someone is ready to talk or who they will feel most connected to. It could be in the middle of the night when a nurse is checking pain levels, after an unexpectedly difficult visit from a family member or with the cleaner where a casual chat turns into something deeper. McSherry^25^ advocates for ‘inter/intra-disciplinary’ spiritual care rather than ‘belonging’ to any particular professional group to ensure integrated spiritual care. These conversations can be challenging both personally and professionally requiring the listener to stand back from their own preferences and experiences to truly and deeply listen to the feelings, desires and pain of the other. Research in a palliative care setting found that providing emotional containment and reflective space for colleagues was identified as important for the multidisciplinary team.^26^

Social workers are ideally placed to help generate interdisciplinary teams that can provide a shared capacity to reflect on spirituality and so create a culture where all team members are more able to respond to people’s needs and preferences. More specifically, social workers using critical spirituality could foster valuing of the spiritual within the social context for individuals and families as well as workers. Critical spirituality as a framework identifies key themes for integrating the spiritual in practice: understanding the self in the context of social structures, the influence of history, the value of relatedness and community and the importance of socially just action and the interconnectedness of all beings.^7^ Combining the critical and spiritual encourages integrating understanding of what is meaningful with how our individual and collective reactions to this are influenced by the context in which we live and our experiences of it. The key themes are derived from complementary theories. First, shared First Nations understandings of the world: awareness of the interdependence of all beings, animate and inanimate^27^ and the importance of the natural world for health and well-being.^28^ Connections to community are a vital part of this^29^ and spirituality embodies ‘a reverence for life as it is. . . a mixture of good and bad, of suffering and joy, and it is celebrated as sacred’.^30^ Finally, awareness of history and social context is key both in giving a sense of belonging but also in recognising the impact of colonisation and need for structural change.

Critical perspectives are based on an analysis of how power is created and maintained socially and structurally (which includes an understanding of personal beliefs and agency in this context) and, therefore, how social inequalities are maintained.^31^ Implicit in this, is challenging assumptions that are incongruent with a social justice perspective, seeking a ‘more just, peaceful, convivial and caring society’.^32^ This includes questioning whether an organisation or community’s policies and practices contribute to unequal or unfair practices.^33^ Combined with postmodernism, critical theory opens up other ways of seeing the world, seeing ‘how structures dominate, but also in how people construct and are constructed by changing social structures and relations, recognising that there may be multiple and diverse constructions of ostensibly similar situations’.^34^ Being critically reflective is an integral aspect of critical spirituality: a process that enables understanding how assumptions and values can be unconsciously embedded in practice in ways that may or not be empowering.^35^ Green social work reinforces noticing the impact of the environment and the interdependence between all beings^36^ and the value of having local Indigenous and community knowledge.^37^ A relational perspective provides the processes needed to embed all of these: finding ways for people to safely express confronting feelings and to move forward reflectively to deeper understanding of what is happening for individuals and groups in ways that lead to constructive and empowering change. A connecting theme here is recognising ‘the important connections between the intrapsychic, interpersonal and broader social contexts in which they are embedded’.^38^

How then can these aspects of critical spirituality be useful in palliative care? What difference could this, a social work perspective, make to people like Cathy?

Social work training in critical theory fosters the capacity to stand back and see the bigger picture, to ask what is influencing what is happening here? What assumptions, or what values and beliefs are institutionalised in the systems we are working in and how are these in turn influenced by the society in which we live? Related to this, social work training includes an expectation of advocacy for broader policy change. This encourages social workers in their work with individuals and families directly and in their broader contexts to seek change. It might be, for example, that the way a palliative care organisation allows visits from family reflects societal norms about who can be a close family member. This might mean that some individuals feel isolated while dying. A social worker might question these expectations from a structural perspective seeking to change the ‘rules’ rather than persuading the individual to accept them. Critical spirituality in the form of reflecting critically on practice can be challenging for individuals and organisations revealing institutional issues that are undermining practice.

The critical lens also prompts workers to be aware of the assumptions that they have internalised from the social context: their family background, their particular experiences and the influence of the prevailing but also changing broader culture over time. This blends with professional values and knowledge, which may or may not reinforce personal experience and beliefs. Social workers frequently use critically reflective processes in individual, peer or group supervision to foster understanding of the influence of their own experiences and beliefs.^31,39^ This awareness also enables social workers to be conscious of the internalised assumptions of those they are working with, their colleagues as well as those who are dying and their families. These may be expressed consciously or unconsciously, with some values and assumptions so deeply held it is challenging to surface and express them.

An example: Zoe, a social worker, came to visit a client, Tim, and found Clare, the occupational therapist trying to persuade him to eat. Tim’s attempts to say he was ready to die so he didn’t want food were ignored and Clare left still expressing frustration partly because Tim’s family also found his not eating challenging and had asked staff to ‘make’ him eat. When Zoe explored Clare’s perspective with her, it was clear her assumption was *it is better to live than to die no matter what*. When she attempted to help Tim explain his assumption of *I know what’s best for me and it’s time to go, I’ve had enough*, Clare felt he was giving up and should try harder for his family’s sake. When Clare later reflected on this in peer group supervision, she could see that she was influenced not only by a family and cultural belief *you should always persevere*, but also by the general social expectation: *you should never give up, that’s being a ‘loser’*. This was reinforced for her by her belief as a Christian that *suffering is part of life and should simply be endured*. Once Clare could name her assumptions and where these came from, she was able to accept Tim’s right to make his own decisions and she and Zoe talked with Tim and his family together. This also demonstrates social workers’ awareness of power and how powerless those in palliative care may feel. Tim was exerting power in the only way he could: refusing to eat. Clare was exerting her power as a professional to try to persuade him to keep living. Zoe was able to act as a mediator to ensure both perspectives were heard and respected.

Cathy wanted this kind of deeper exploration of some aspects of her personal history. Wiebe^17^ points out that a common theme at the end of life ‘is dealing with regrets’ and that social workers can encourage people to express these, finding forgiveness of others and themselves. Critical spirituality can also remind us of how these connect to related personal and broader structural history. Cathy’s experience was of being deeply committed to her religious tradition and so the organisation she worked for auspiced by that tradition. Her personal experience meant that she had a strongly developed sense of social justice and advocacy which was part of the organisation’s espoused values. However, she felt that she was seen as an eccentric outsider who took these values ‘too far’, advocating for what she saw as inclusive change that was seen as undermining traditional processes. Ultimately, she was made redundant, which remained a very painful memory. When she raised this issue with the pastoral care worker, she felt the worker was comforting her, but not able to help her see this experience differently. When I used a critical spirituality stance as her default social worker, she was able to explore the different values and assumptions being expressed: her own related to power of *I must speak up for those who are being marginalised as I have been* versus others in the organisation: *I am feeling marginalised by Cathy’s suggested changes that undermined the comforting processes I am used to.* She could also acknowledge there were some who supported her but felt they needed also to recognise the struggles of others facing change. As she talked more about what else was happening, she could see that there were broader financial and social issues, and changes in government policy affecting the organisation – the redundancy wasn’t only about her. She could see that she had moved unconsciously to a new assumption: *I can do this in another way* and had taken her religious values into her local community in a more secular way, where they had been appreciated. This process helped her let go of much of the pain of this experience and to see it from a new perspective: *my way works equally well and sometimes better*. As Cathy’s ‘worker’ I could see that reflecting on the broader context, a key aspect of critical spirituality, enabled Cathy to see how this influenced her experience and to let go of some past unhelpful assumptions and to articulate and affirm more helpful new or unconscious assumptions. This fostered a sense of peace in the process of dying.

This took Cathy to explore the next aspect of critical spirituality: the importance of community connection. She affirmed how important the local community had become to her, like family, a source of shared interests and interconnectedness. Cathy had helped generate a culture of mutual support in the community, both for herself and others. Enabling her to maintain contact with her community was something she felt the palliative care team did well. However, she was conscious that this worked for her as a white woman with a small number of family and friends/community members only wanting to see one person at a time. She wondered how the staff would manage larger more exuberant groups of families and communities. Social workers can advocate for such community connections, including the use of volunteers who may have more time to build relationships and create community within the palliative care setting.

What Cathy also wanted and valued from most of the palliative care team was the importance of relatedness, an ability to see her as a whole person, not just an illness.^24^ This was combined with what First Nations people call deep listening: listening for the deeper meaning and connections for each individual to people, community and to the natural world. This includes the humility of respecting the fundamentally equal value of each person^40^ and the ability to wait, to shift from doing to being mode. Social workers can advocate for time for this using interpersonal skills to generate a culture or spirit of openness, asking what is meaningful for this particular person? It may be that the professional and the dying person have quite different and even contradictory religious practices when it comes to dying and death, which means each feels alienated from the other^41^ instead of having a celebratory attitude to diversity.

For Cathy, most palliative care professionals felt ‘naturally’ warm and empathic and she found she could often have real conversations. However, she was surprised that some team members found her mix of spiritual and religious practices difficult including her combination of Buddhist and Christian symbols. She raised this with the pastoral care worker who explained that for some there was an expectation of having one consistent way of being or practising: being religious or not, spiritual or not. In this particular hospice, training was planned to generate more self-awareness and understanding of spiritual diversity, but staff often didn’t feel confident at this stage. Cathy didn’t feel she had the energy to challenge such modernist assumptions and quickly tuned into who to ask for what she wanted or what kinds of conversations to have with which person. Social workers using a critical spirituality perspective would advocate for the team to recognise the diversity of spiritual and religious expression. Gardner^42^ found that critically reflective training for palliative care teams in spiritual care expanded understanding of spirituality with one participant commenting ‘helpful to understand how spiritual care crosses all areas and is part of all of us’. For one exercise, participants chose a particular card with an image and a word relating to spirituality. Sharing these fostered seeing differences and valuing the complexity of each person and their context.

Finally, a critical spiritual perspective acts as a reminder of the interconnectedness and holistic nature of all beings. When patients in a rehabilitation ward in a rural hospital in Australia were asked what kept them going, they responded with stories of fishing, missing their cows and contact with nature.^24^ Similarly, being able to see trees and gardens or sit in a courtyard with trees and flowers was deeply nurturing. These were important to Cathy who was a dedicated gardener; what would have helped and wasn’t seen as possible was having her dog visit. The music therapist provided a different form of meaning and connection to the transcendent for many. Social workers need to work both with the team and the organisation to make these varieties of connectedness more possible.

Truly embedding a critical spirituality approach requires commitment: it is partly about a cultural shift in how to perceive working with those who are dying and their families. Structures are needed to foster understanding of what the critical means here and how to engage with broader structural understandings. Training for all staff and volunteers that makes explicit the underlying theories and practising the process helps but generally, workers want ongoing mutually supportive relationships to embed this.^42^ This means active support from the organisation’s management including recognition that workers engaged in being critically spiritual and so critically reflective will inevitably challenge existing practices and policies and that this is part of a healthy learning organisation.^43^

The aim ultimately is for this to become an integral part of the organisation, like critical reflection ‘rather than something you do, *being* critically reflective becomes embedded into the culture’.^39^ Similarly, critical spirituality would become part of how the team operates. Supervision in various forms can help to create this culture – individually, with a peer, formally or informally, or in peer supervision groups from individual or mixed disciplines. There are significant advantages in the more egalitarian nature of peer supervision where workers hearing the different perspectives of others, become more open to challenging their own assumptions. In a large health care agency, for example, using peer supervision for allied health, workers shared specific experiences that they found troubling or puzzling and enabled each other to name feelings and underlying assumptions and values that enabled them to understand their experience more deeply in a way that often led to changed practice.^44^ Participants also found that they shared similar feelings and challenges to other disciplines, understood better their different perspectives and became more able to address conflicts within and outside the group.^44^

Social workers are ideally placed to embed the ideas and practices of critical spirituality across palliative care teams. A critical approach is fundamental to social work training and reinforces valuing diversity and seeking socially just change. Social work as a profession names and enables seeing the connections between individuals and the influence of their history and their personal and broader social context. Critical spirituality is an approach that also makes more explicit the inclusion of the spiritual for social workers. Related values and processes from First Nations perspectives, green social work and relational theories are increasingly evident in social work training and together foster awareness of history and social context. Critical spirituality reinforces social workers advocating for change as needed at all levels: individual, organisational and in social structures. Naming critical spirituality as integral to practice can create a culture enabling all those involved in receiving as well as giving palliative care in understanding more deeply the importance of meaning and related assumptions and values. This can foster the interconnections and processes needed at end of life.

## Declarations

**Ethics approval and consent to participate:** Not applicable.

**Consent for publication:** Not applicable.

**Availability of data and materials:** Not applicable.
